# Sodium Ingestion Improves Groundstroke Performance in Nationally-Ranked Tennis Players: A Randomized, Placebo-Controlled Crossover Trial

**DOI:** 10.3389/fnut.2020.549413

**Published:** 2020-09-22

**Authors:** Edward H. Munson, Samuel T. Orange, James W. Bray, Shane Thurlow, Philip Marshall, Rebecca V. Vince

**Affiliations:** ^1^Department of Sport, Health and Exercise Science, Faculty of Health Sciences, University of Hull, Hull, United Kingdom; ^2^School of Biomedical, Nutritional, and Sport Sciences, Faculty of Medical Sciences, The Medical School, Newcastle University, Newcastle upon Tyne, United Kingdom; ^3^Institute for Sport, Physical Activity and Leisure, Leeds Beckett University, Leeds, United Kingdom

**Keywords:** exercise physiology, tennis, performance nutrition, sodium, fluid balance, hydration

## Abstract

This study examined the dose-response effects of ingesting different sodium concentrations on markers of hydration and tennis skill. Twelve British nationally-ranked tennis players (age: 21.5 ± 3.1 years; VO_2peak_: 45.5 ± 4.4 ml^.^kg^.^min^−1^) completed four identical in-door tennis training sessions in a cluster randomized, single-blind, placebo-controlled, crossover design. Twenty-minutes prior to each training session, participants consumed a 250 ml sodium-containing beverage (10, 20, 50 mmol/L) or a placebo (0 mmol/L), and continued to consume 1,000 ml of the same beverage at set periods during the 1-h training session. Tennis groundstroke and serve performance, agility, urine osmolality, fluid loss, sodium sweat loss and perceptual responses (rating of perceived exertion (RPE), thirst, and gastrointestinal (GI) discomfort) were assessed. Results showed that ingesting 50 mmol/L sodium reduced urine osmolality (−119 mOsmol/kg; *p* = 0.037) and improved groundstroke performance (5.4; *p* < 0.001) compared with placebo. This was associated with a reduction in RPE (−0.42; *p* = 0.029), perception of thirst (−0.58; *p* = 0.012), and GI discomfort (−0.55; *p* = 0.019) during the 50 mmol/L trial compared with placebo. Linear trend analysis showed that ingesting greater concentrations of sodium proportionately reduced urine osmolality (β = −147 mOsmol/kg; *p* = 0.007) and improved groundstroke performance (β = 5.6; *p* < 0.001) in a dose response manner. Perceived thirst also decreased linearly as sodium concentration increased (β = −0.51; *p* = 0.044). There was no evidence for an effect of sodium consumption on fluid loss, sweat sodium loss, serve or agility performance (*p* > 0.05). In conclusion, consuming 50 mmol/L of sodium before and during a 1-h tennis training session reduced urine osmolality and improved groundstroke performance in nationally-ranked tennis players. There was also evidence of dose response effects, showing that ingesting greater sodium concentrations resulted in greater improvements in groundstroke performance. The enhancement in tennis skill may have resulted from an attenuation of symptomologic distracters associated with hypohydration, such as RPE, thirst and GI discomfort.

## Introduction

Tennis is a repetitive sprint sport, characterized by intermittent bouts of high-intensity exercise interspersed with periods of rest or low-intensity activity (1). The duration of a tennis match often exceeds 1 h and in some cases can last more than 5 h (2). Players also require a combination of fine and gross motor skills, agility and power to execute sport-specific tasks such as serves, groundstrokes, and volleys. Due to the high physiological demands, often under challenging environmental conditions, sweat losses during tennis match-play can be significant, with mean sweat rates of 0.6–2.6 L/h reported in the literature (3). This can lead to hypohydration (loss of body water) unless appropriate hydration strategies are implemented.

In addition to water, sweat contains substantial but variable amounts of sodium. Sweat sodium losses during tennis match-play are generally between 20 and 80 mmol/L (4–7). Excessive sodium lost through sweat, particularly when combined with over-drinking hypotonic beverages, reduces plasma sodium concentration leading to hyponatremia when plasma concentration is <135 mmol/L (8). This is problematic for athletes because sodium is an essential electrolyte that helps with fluid retention, cognition, muscle contraction, and nerve conduction (9–12), all of which are critical for the execution of technical skills in tennis.

The American College of Sports Medicine (ACSM) recommend that sodium should be ingested during exercise when large sweat sodium losses occur (13). The ACSM also suggest that sodium consumed in pre-exercise beverages may help athletes achieve euhydration prior to exercise (13). However, the evidence supporting this recommendation is limited. A recent systematic review (14) found that only one of the five included studies reported a significant benefit of sodium ingestion on endurance performance. In addition, four of the five included studies were conducted outdoors (14), meaning that the findings could have been confounded by factors such as ambient temperature, relative humidity, wind speed and direction. Furthermore, it is unknown whether sodium ingestion influences tennis-specific skill, which involves a combination of physical, technical, and cognitive factors.

There are currently no guidelines on the specific quantity of sodium ingestion required to optimize technical skills in sport, nor whether there is a dose-response relationship. Previous research has shown that consuming fluids with a higher sodium concentration reduces the occurrence of hyponatremia during prolonged exercise compared with fluids with a lower sodium concentration (15). However, this may not translate into improved sports performance (15). There is a need for further well-controlled studies to inform sports nutritional guidelines and sodium replacement strategies. Therefore, the primary purpose of this study was to examine the dose-response effects of ingesting different sodium concentrations on hydration status, fluid balance, agility, perceptual responses and tennis skill performance in British nationally-ranked tennis players.

## Materials and Methods

### Study Design

This study used a cluster randomized, single-blind, placebo-controlled, crossover design. Participants completed two familiarization trials and an incremental cardiopulmonary exercise test (CPET), followed by four identical tennis training sessions that were separated by 7 days. Twenty-minutes prior to each tennis training session, participants consumed a 250 ml sodium-containing beverage (10, 20, 50 mmol/L) or a placebo, and continued to consume 1,000 ml of the same beverage at set periods during the training session. The order of the beverages was randomized but was not counter-balanced i.e. all participants received the beverages in the same order. Body mass (kg) and urine osmolality (UOsm) were measured immediately before and after training sessions to assess fluid loss and hydration status, respectively. Groundstroke and serve performance, agility, rating of perceived exertion (RPE), thirst, and gastrointestinal (GI) discomfort were recorded during the training sessions, whilst sweat sodium concentration and total sweat sodium loss were assessed immediately afterwards.

### Participants

Twelve nationally-ranked tennis players [mean (SD) age: 21.5 ± 3.1 years; body mass: 71.5 ± 7.1 kg; height: 178 ± 5 cm; peak oxygen consumption (VO_2peak_): 45.5 ± 4.4 ml^.^kg^.^min^−1^] were recruited from the University of Hull Men's 1st Tennis Team and volunteered to take part in this study. All participants were currently ranked between 50 and 1,000 in Great Britain based on Lawn Tennis Association (LTA) national rankings, and regularly competed in LTA Grade 3 tournaments (high-level regional standard). Participants were part of the same training group and informed of the experimental procedures prior to signing an institutionally approved informed consent document to participate in the study. Ethical approval for the study was granted by the Sports, Health and Exercise Science Ethics Committee at the University of Hull.

### Procedures

#### Familiarization Sessions and Cardiopulmonary Exercise Test (CPET)

The week prior to the first experimental training session, participants completed two familiarization trials and an incremental CPET on separate days. Participants initially completed a medical questionnaire and had their body mass, height, resting blood pressure and heart rate recorded. The incremental CPET was then performed on a motorized treadmill (Cosmos Pulsar, H/P Cosmos, Nussdorf-Traunstein, Germany) to characterize participants' cardiopulmonary fitness at baseline. The CPET protocol began with a treadmill speed of 8 km^.^h^−1^ at a constant incline of 1.5%, which increased in speed every 3-min by 2 km^.^h^−1^ respectively, until volitional exhaustion. Exhaustion was defined as an inability to maintain the required running speed despite strong verbal encouragement. Breath-by-breath data (Oxycon Pro, CareFusion, Hoechberg, Germany) were recorded throughout and averaged per minute before interpretation. VO_2peak_ was determined as the highest VO_2_ attained during the final 30-s of the CPET. The familiarization sessions were identical to the tennis training sessions apart from that no data were collected and no restrictions were implemented.

#### Tennis Training Session

Participants consumed 500 ml of water 4-h before each tennis training session to ensure they started in a euhydrated state. All training sessions lasted 1-h and took place across two in-door tennis courts inside a three-court facility. Participants were instructed not to perform moderate- to vigorous-intensity exercise for at least 48 h prior to each experimental session. Sessions were performed at the same time of day (7:00 p.m.), each separated by exactly 7 days. Ambient temperature (16.5 ± 1.1°C) and humidity (30 ± 7.6%) were maintained using the indoor air conditioning unit and were monitored remotely throughout using a wireless weather station (BAR816HG, Oregon Scientific, Tualatin, Oregon). This reflects the climate set in UK indoor tennis facilities. Participants wore only a t-shirt, shorts, socks, and shoes during the training sessions. Participants were provided with a 3-min break every 15-min throughout each training session, to allow them to consume the sodium-containing beverage (or placebo).

Training sessions began with a standardized 10-min dynamic warm-up, followed by a 15-min tennis-specific warm-up, prior to the main training session. The tennis-specific warm-up consisted of paired stroke shot rallying (2-min), volleys (1-min), and serve practice (2-min) across half a court, followed by 2 × 5-min half-court matches. Standard tennis rules applied during the half-court games, apart from that players could use the tram-lines during the point. The same opponents were used in each training session, which were matched as closely as possible based on their current LTA ranking. The stroke performance test involved two technical tennis drills; groundstrokes and serves. First, participants hit 20 forehands and 20 backhands cross-court, aiming for 3 × 3 m^2^ target areas in both opposite corners of the court, which were marked with lines. They performed four shots (2 forehands and 2 backhands), jogged round to collect the four balls and returned for another four shots until 40 had been completed. Participants were encouraged to perform “attacking” shots to the best of their ability and performed the drill in the same order each time, waiting at the back of the court until it was their turn to perform the drill. An LTA qualified coach was stood on the opposite side of the court at the net and threw the ball underarm to the participants in the same place for the forehand and backhand (in the corner between the base-line and tram-line) marked out by a 1 × 1 m^2^ lined target. After a player sprinted to perform a shot, they were allowed 2 s to return to the center of the baseline ready to perform the next shot emphasizing the need for a quick acceleration and deceleration before and after each shot. The ball bounced once before being struck by the participant. This drill was performed on two tennis courts with six participants on each court. The same coaches threw the balls for each participant during all experimental sessions. Participants then performed 10 serves from the right and 10 serves from the left, aiming for a 2 × 4.6 m^2^ area at the top of both service boxes marked with lines. They were allowed 30 s between serve attempts. To minimize learning effects, the tennis training session involved drills that the participants were highly accustomed to performing during prior routine training sessions. After finishing the tennis drills, participants completed a Pro-Agility test (see outcome measures below).

#### Sodium-Containing Beverages

Twenty-minutes before the start of each experimental session, participants consumed a 250 ml beverage containing either 0 (placebo), 10, 20, or 50 mmol/L of sodium (Light Gray Celtic Sea Salt, Selina Naturally, Arden, NC) in a cluster randomized, non-counterbalanced order. Participants then continued to consume 1,000 ml of the same beverage at set 3-min intervals every 15-min during the training session. These intervals were chosen to replicate the frequency of rest intervals in competition. The volume of fluid consumed during the 3-min intervals was *ad libitum*, but participants were instructed to consume the entire 1,000 ml beverage by the end of the session, which was confirmed by a research team member. The order of the experimental sessions for all participants was: 10, 50, 0, and 20 mmol/L. Commercially available sports drinks generally provide ~20 mmol/L of sodium per serving (16). Hence, the concentrations of sodium used in this study reflect lower, similar and higher concentrations for comparison. The flavor of each solution was masked with zero-calorie flavoring (Flavdrops^TM^, MyProtein, Warrington, UK), and participants were blind to the beverage they received. As per the participant information sheet, participants were told that the researchers were testing the effect of four different sports drinks on performance. They were informed that the highest sodium concentration of any drink was 50 mmol/L, but they were unaware that the sodium content of the drink differed nor that the researchers were specifically testing for the effects of sodium. All participants confirmed that they did not know which beverage they were receiving after completing the study. They were not permitted to spit out any of the liquid or pour it onto their hair or face. Beverages in all four experimental conditions were made with the same brand and batch of still water (ASDA Still Natural Mineral Water, Asda, Leeds, UK) to ensure consistency of the water mineral content. The mineral concentration of the water was as follows: Calcium, 0.23 mmol/L; Magnesium, 0.08 mmol/L; Potassium, 0.051 mmol/L; and Sodium, 0.4 mmol/L. The sodium concentration of each beverage was confirmed prior to training sessions using the B-722 LAQUAtwin Sodium Ion Meter (Horiba, Kyoto, Japan), which was calibrated before every session using a standard solution containing 150 parts per million (ppm) of sodium.

#### Dietary Analysis

Participants completed a 48-h record of their food and fluid intake before each tennis training session. We instructed participants to maintain their habitual, eucaloric diets throughout the study period, and to consume the same foods/fluids for 48-h prior to each session. Participants also refrained from consuming alcohol or caffeine for 48-h before training sessions. Diet records were analyzed for sodium content using the smartphone application MyFitnessPal (Under Armor, Baltimore, MD).

### Outcome Measures

#### Hydration Status

Change in hydration status from pre- to post-session was determined with UOsm. After consuming the 250 ml beverage 20-min prior to the tennis training session, participants emptied their bladders and provided a midstream urine sample directly into 30 ml clear, plastic, sterile container. A second sample was collected post-session. Urine samples were assessed for UOsm within 10-min of collection using a portable osmometer (Osmocheck, Vitech Scientific, West Sussex, UK) that has previously been validated (17). The osmometer provides a measurement range of 0–1,500 mOsmol/kg and was calibrated using distilled deionised water before each session.

#### Sweat Sodium Concentration

Immediately before participants began the tennis training sessions, four absorbent sweat pads (8 × 6 cm; Adhesive Dressing, Boots, Nottingham, UK) were applied to four sites of the body: (1) on the posterior midline of the right forearm, equidistant between the antecubital fossa and wrist joint, (2) the midline of the widest circumference of the right calf, (3) on the anterior midline of the right thigh, equidistant between the patella and greater trochanter, and (4) 5-centimeters lateral from the third lumbar spine vertebra (L3). These sites were chosen to align with previous research (5) and because they are highly correlated to whole-body sweat sodium loss (18) and provide minimum disruption of tennis performance (5). Prior to application, each patch site was shaved with a handheld razor, cleaned using an alcohol wipe, washed with deionised water and dried with a clean, electrolyte free swab gauze (Boots). Immediately post-session, the sweat pads were removed using a pair of sterile forceps (a new pair for each patch) and individually placed inside the barrel of a 10-ml plastic syringe (King Scientific, Liversedge, UK). Deionised water (5-ml) was added to the patch to ensure a sufficient volume of solution was obtained for analysis. The syringe plunger was then depressed to compress the pad and obtain a minimum 5-ml sample of sweat. This sample was immediately analyzed for sodium concentration (mmol/L) by the B-722 LAQUAtwin Sodium Ion Meter, which corrected for the 5 ml of deionised water previously added. This method is a valid field technique of estimating sodium concentration (19). Patches were not assessed for background sodium. Whole-body sweat sodium concentration was determined through a validated area weighted mean of four skin regions (18) as adapted by previous tennis sweat sodium research (5, 18). Mean whole-body sweat sodium concentration = 28.2% calf + 28.2% scapula + 11.3% forearm + 32.2% thigh.

#### Fluid and Sweat Sodium Loss

Fluid loss was estimated from the pre-post change in body mass after correcting for the 1,000 ml of fluid consumed throughout the 1-h training session. Body mass was measured to the nearest 0.1 kg using a calibrated digital scale (seca 813, SECA, Birmingham, UK). Participants wore only underwear and removed all jewelry. Body mass was then re-assessed within 5-min of the conclusion of the training session, before participants had urinated/defecated/consumed food or liquid, and after sweat pads had been collected from the skin sites and participants thoroughly towel dried their bodies. In line with previous research (5, 18, 20, 21), loss of mass was not corrected for respiratory water loss or loss due to substrate exchange because this would be impractical for practitioners to include when assessing fluid requirements (22). Total sweat sodium loss (g) was calculated by multiplying the volume of fluid loss (L) by the molecular mass of sodium (22.99 g/mol) and the concentration of whole-body sweat sodium loss (mmol/L) (5).

#### Perceptual Responses

Rating of perceived exertion (RPE), gastrointestinal (GI) discomfort, and perceived thirst data were collected during each 15-min break during the 1-h training session and after the session had finished (total of 4 times). A 10-point Borg scale (arbitrary units [AU]) was used to collect RPE data (23). Thirst and GI discomfort were also rated on a 10-point Likert scale (AU) ranging from “No discomfort” and “Not thirsty” to “Unbearable” and “Excessive thirst,” respectively. Participants were formally familiarized with the scales during the two prior familiarization sessions. The four scores in each outcome were averaged prior to analysis to reduce the number of statistical comparisons made.

#### Stroke Performance

The number of forehand strokes, backhand strokes, and serves to land in the pre-specified target areas during the tennis training session were recorded. Forehand and backhand stroke scores were combined to provide an overall tennis groundstroke score (maximum of 40). A point was given if any part of the ball touched the line encompassing the target area. The drills were recorded by a 50 Hz video camera (Panasonic Lumix DC-TZ200), with the videos being viewed post-session to determine the number of successful attempts.

#### Pro-Agility Test

Agility performance was assessed with the Pro-Agility test. A distance of 9.14 m was measured and marked with a line on the tennis court. Participants began in a neutral 3-point position with feet placed equally either side of the midline. After a countdown of “one, two, three, go” by the researcher, participants turned and ran 4.57 m to their left and touched the line with their left hand, then ran 9.14 m to the right whilst touching the line with their right hand, then ran back 4.57 m through the midline. Time began with initial movement and ended when the participant crossed the midline a second time, covering a total distance of 18.28 m. Two researchers recorded time with a stopwatch, with the average time recorded to the nearest to the nearest 0.01-s. Three trials were performed, separated by 3-min of rest, with the fastest time used for analysis. Participants completed the test in the same order during each session.

### Statistical Analyses

Data were analyzed in R (R Foundation for Statistical Computing, Vienna, Austria). Descriptive statistics are reported as mean ± SD or median (interquartile range). Paired *t*-tests with a Bonferroni correction were used to compare pre-trial UOsm and body mass between conditions. Differences in study outcomes between conditions were assessed with a multilevel linear model. Condition was entered into the model as a fixed factor with four levels (0, 10, 20, or 50 mmol/L sodium) and participants were entered as a random factor with individual intercepts. For pre-post changes in UOsm and body mass, the pre-session value was also entered into the model as a covariate. If there was a significant main effect of condition (i.e. if the inclusion of “condition” in the model significantly improved the model fit), the data were further explored using three non-orthogonal contrasts; where the 10, 20, and 50 mmol/L conditions were separately compared to the placebo condition. The contrasts were adjusted for multiple comparisons using a Bonferroni correction, and mean differences with their 95% confidence intervals (CIs) from the comparisons are presented. Subsequently, we conducted a linear trend analysis using the stats::contr.poly() function in R to investigate whether there was a linear dose response effect. Statistical significance was set at a two-tailed *p* < 0.05.

## Results

Descriptive statistics are presented in Table 1. For outcomes with a significant main effect of condition, follow-up comparisons and linear trend analyses are presented in Table 2.

**Table 1:** Descriptive statistics of outcomes in each experimental condition (mean ± SD or median [IQR]).

	**50 mmol/L**	**20 mmol/L**	**10 mmol/L**	**Placebo**	**Main effect of condition (*p*)**
**Body mass (kg)**					
Pre-trial	71.9 ± 7.6	71.9 ± 7.2	72.5 ± 7.3	72.1 ± 7.1	–
Post-trial	72.5 ± 7.5	72.3 ± 7.1	72.9 ± 7.3	72.5 ± 7.1	–
Change	0.55 ± 0.14	0.48 ± 0.22	0.45 ± 0.18	0.37 ± 0.15	0.08
Fluid loss (L/hour)^a^	−0.45 ± 0.14	−0.52 ± 0.22	−0.55 ± 0.18	−0.63 ± 0.15	0.08
**Urine osmolality (mOsmol/kg)**					
Pre-trial	635 [500, 745]	490 [380, 743]	485 [338, 663]	405 [210, 743]	–
Post-trial	395 [345, 610]	430 [388, 723]	480 [368, 708]	645 [388, 758]	–
Change	−210 (−308, −48)^*^	−25 (−105, 63)	55 (10, 185)	90 (−40, 178)	0.015
**Sweat Na**^**+**^ **(mmol/L)**					
Whole-body	69.7 ± 9.4^*^	67.3 ± 8.6^*^	54.9 ± 8.0^*^	61.3 ± 11.1	<0.001
Total sweat Na^+^ loss (g)^b^	−0.73 ± 0.30	−0.79 ± 0.36	−0.70 ± 0.27	−0.89 ± 0.26	0.37
**Perceptual responses**					
RPE	3.2 ± 1.1^*^	3.9 ± 0.7	3.8 ± 1.1	3.5 ± 1.1	0.019
GI discomfort	2.4 ± 0.9^*^	3.6 ± 1.2	3.1 ± 0.8	2.6 ± 0.9	0.002
Thirst	2.8 ± 0.9^*^	3.8 ± 0.7	3.0 ± 0.8	3.8 ± 1.2	0.001
**Tennis skill and agility**					
Successful stroke attempts	22.2 ± 5.7^*^	15.3 ± 4.8	16.3 ± 4.6	13.5 ± 5.0	<0.001
Successful serve attempts	10.8 ± 2.4	10.6 ± 1.7	10.6 ± 2.8	10.5 ± 2.2	0.99
Pro-Agility test (s)	4.5 ± 0.1	4.6 ± 0.1	4.5 ± 0.2	4.5 ± 0.1	0.11

*GI = gastrointestinal; p = p-value; RPE = rating of perceived exertion*.

a *Fluid loss was calculated as: pre-post change in body mass (kg)−1,000 (ml) to correct for the 1,000 ml of fluid consumed at set periods throughout the 1-h tennis training session*.

b *Total sweat sodium loss was calculated as the product of fluid loss, the molecular mass of sodium, and whole-body sweat sodium (Na^+^) concentration*.

* *Statistically different from placebo (p < 0.05)*.

**Table 2:** Mean differences (95% CI) in outcomes between experimental conditions.

	**50 mmol/L vs. placebo**		**20 mmol/L vs. placebo**		**10 mmol/L vs. placebo**		**Linear trend**	
	**Mean (95% CI)**	***p***	**Mean (95% CI)**	***p***	**Mean (95% CI)**	***p***	**β (95% CI)**	***p***
Change in urine osmolality (mOsmol/kg)	−119 (−227, −11.9)	0.037	−11.0 (−116, 94.2)	1.0	51.0 (−54.2, 156)	0.76	−147 (−248, −45.6)	0.007
Whole-body Sweat Na^+^ (mmol/L)	6.4 (3.0, 9.9)	<0.001	4.0 (0.51, 7.5)	0.028	−8.4 (−11.9, −4.9)	<0.001	8.5 (4.3, 12.6)	<0.001
RPE	−0.42 (−0.78, −0.05)	0.029	0.33 (−0.03, 0.70)	0.11	0.18 (−0.19, 0.54)	0.77	−0.18 (−0.57, 0.20)	0.35
GI discomfort	−0.55 (−1.0, −0.09)	0.019	0.66 (0.20, 1.1)	0.005	0.18 (−0.28, 0.64)	1.0	−0.07 (−0.60, 0.45)	0.79
Thirst	−0.58 (−1.0, −0.13)	0.012	0.45 (0.00, 0.90)	0.062	−0.33 (−0.77, 0.12)	0.26	−0.51 (−1.0, −0.03)	0.044
Groundstroke performance	5.4 (3.1, 7.6)	<0.001	−1.5 (−3.8, 0.72)	0.33	−0.54 (−2.8, 1.7)	1.0	5.6 (3.3, 7.9)	<0.001

*95% CI = 95% confidence interval; GI = gastrointestinal; p = p-value; RPE = rating of perceived exertion*.

### Hydration Status and Fluid Loss

There were no significant differences in pre-trial UOsm or body mass between conditions (*p* > 0.05; Table 1), which suggests that participants were in a similar hydrated state before each trial. There was no significant effect of condition on pre-post change in body mass (*p* = 0.076) or fluid loss (*p* = 0.080). However, there was a significant main effect of condition for the pre-post change in UOsm (Table 1). Contrasts showed that the reduction in UOsm was significantly greater in the 50 mmol/L trial compared with placebo (−119 [−227, −11.9] mOsmol/kg, *p* = 0.037). Furthermore, trend analysis showed a significant linear relationship between sodium ingestion and UOsm, demonstrating that as the dose of sodium increased, UOsm decreased proportionately (β = −147 [−248, −45.6] mOsmol/kg; *p* = 0.007).

### Sweat Sodium Concentration

There was a significant main effect of condition on whole-body-sweat concentration (Table 1). Both the 50 mmol/L (6.4 [3.0, 9.9] mmol/L; *p* < 0.001) and 20 mmol/L (4.0 [0.51, 7.5] mmol/L; *p* = 0.028) trials reported significantly higher whole-body sodium sweat concentrations compared with placebo (Table 2), and there was a significant linear trend between condition and whole-body sweat sodium concentration (β = 8.5 [4.3, 12.6] mmol/L; *p* < 0.001). However, there was no significant effect of condition on total sodium sweat loss (*p* = 0.37). Dietary sodium intake in the 48-h before each trial was not different between conditions (50 mmol/L = 4.4 ± 1.5 g; 20 mmol/L = 3.7 ± 0.66 g; 10 mmol/L = 3.8 0 ± 0.89 g; placebo = 4.0 ± 1.2 g; *p* = 0.20).

### Tennis Skill and Agility Performance

**Figure 1:**
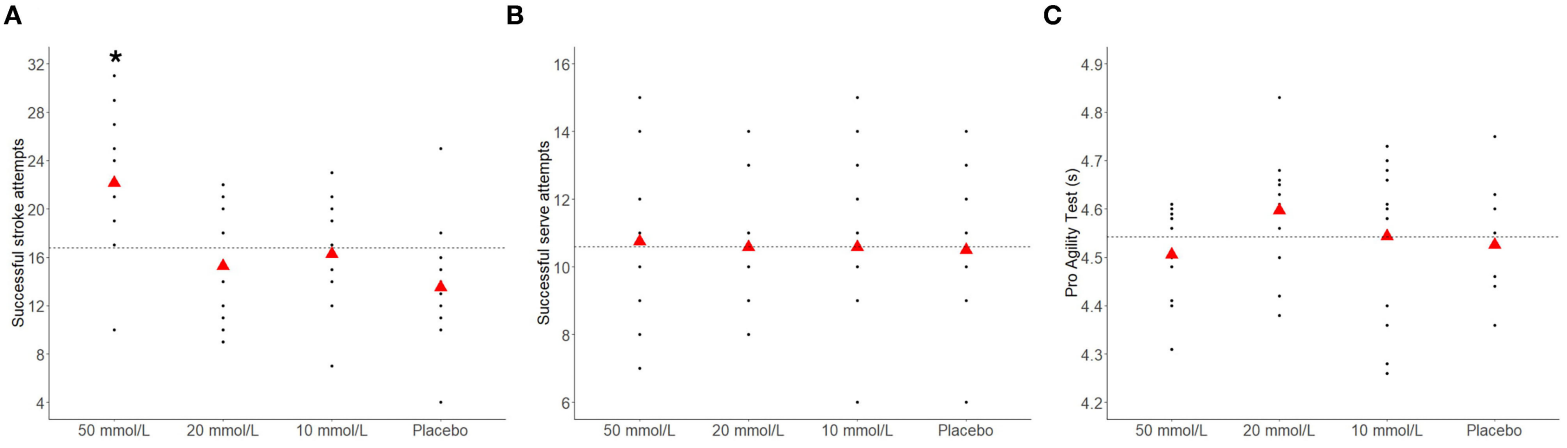
Groundstroke **(A)**, serve **(B)**, and agility **(C)** performance during each experimental condition. The dashed horizontal line represents the mean score across all conditions. *Significantly different to placebo (*p* < 0.001).

Groundstroke, serve, and agility performance are presented in Table 1 and Figure 1. There was a significant main effect of condition for groundstroke performance, with follow-up contrasts showing that groundstroke performance was significantly greater during the 50 mmol/L trial compared with placebo (5.4 [3.1, 7.6]; *p* < 0.001). Further, there was a significant linear relationship between sodium concentration and stroke performance, demonstrating a dose response effect (β = 5.6 [3.3, 7.9]; *p* < 0.001). There was no significant effect of condition on serve (*p* = 0.99) or agility (*p* = 0.11) performances.

### Perceptual Responses

There was a significant main effect of condition on all perceptual responses. Perceived thirst (−0.58 [−1.02, −0.13]; *p* = 0.012), GI discomfort (−0.55 [−1.01, −0.09]; *p* = 0.019), and RPE (−0.42 [−0.78, −0.05]; *p* = 0.029) were all significantly lower in the 50 mmol/L trial compared with placebo (Table 2). Trend analysis also showed that as the beverage sodium increased, perceived thirst decreased in a linear manner (β = −0.51 [−1.00, −0.03]; *p* = 0.044). GI discomfort was greater in the 20 mmol/L trial compared with the placebo condition (0.66 [0.20, 1.11]; *p* = 0.005).

## Discussion

This is the first study to evaluate the effectiveness of consuming different sodium concentrations on markers of hydration and tennis skill performance in nationally-ranked tennis players. The main findings were that 50 mmol/L of sodium ingestion before and during a 1-h tennis training session reduced UOsm and improved groundstroke performance compared with placebo. There was also evidence of dose-response effects, demonstrating that ingesting greater concentrations of sodium promoted proportionately greater improvements in hydration status and groundstroke performance. These results provide novel evidence for sodium consumption as an effective nutritional strategy for enhancing tennis skill.

It is well-established that hypohydration reduces aerobic capacity and cognitive function (24–26). Given that tennis skills are dependent on a combination of physical and cognitive factors, hypohydration may also account for impaired technical skill (3). Indeed, whilst research in tennis is scarce, studies with basketball (27, 28) and soccer players (29) have reported impaired execution of sport-specific skills, although this is not always the case (30, 31). A novel finding of our study was that there was a dose-dependant increase in the effects of sodium on tennis performance in a randomized placebo-controlled, crossover trial. Ingesting 50 mmol/L of sodium before and during a tennis training session improved groundstroke performance compared with placebo. Consuming 50 mmol/L sodium also resulted in a greater UOsm reduction from pre- to post-trial compared with placebo (-119 mOsmol/kg), which is indiciative of a change in hydration status. Accordingly, using UOsm ≥ 700 mOsmol/kg to define hypohydration (25), four of 12 participants in the placebo trial were considered hypohydrated post-session, compared with only one participant in the 50 mmol/L sodium trial. Thus, our findings suggest that the improved groundstroke performance following 50 mmol/L of sodium ingestion was underpinned by changes in UOsm.

Although urine volume was not measured in this study, which could be considered a limitation, it was expected that the 50 mmol/L trial would have produced lower urine volume thus increased UOsm (32). Pre-trial UOsm was insignificant between trials, but values increased numerically higher. As the pre-trial urine sample was collected 20-min after the initial 250 ml bolus had been consumed, the placebo drink may have increased urine output and produced a more dilute urine concentration, potentially affecting the difference between pre-post exercise UOsm. Therefore, measurement of urine volume should be considered in future studies. Furthermore, the 50 mmol/L trial showed the highest pre-trial UOsm reading compared to all other trials (635 mOsmol/kg), suggesting the 50 mmol/L drink could have had a greater hydrating effect due to differences in baseline values. Nevertheless, this difference in UOsm at baseline did not reach statistical significance, and we controlled for differences in baseline values and regression to the mean effects by including the baseline scores as a covariate in the statistical model.

Trend analyses provided evidence for dose-response effects of sodium ingestion on hydration and groundstroke performance. That is, as the concentration of sodium increased from placebo to 10, 20, and 50 mmol/L, UOsm changes and groundstroke performance improved linearly. This result provides further support for our findings and the use of sodium as an ergogenic aid in tennis, and warrants further research evaluating the optimal dose of sodium to enhance sport performance. Sodium consumption may improve hydration status by increasing plasma sodium concentration, which helps retain ingested fluid via osmotic processes. Improved hydration status has a direct effect on physical performance by maintaining plasma volume, body core temperature, muscle perfusion and muscle metabolism, amongst other mechanisms (26, 32). In contrast, there appears to be no clear physiological mechanism by which modest improvements in hydration status might improve sport skill or cognitive function (32). Instead, improved hydration may attenuate the distracting symptoms of hypohydration, such as negative mood state (33), thirst (33), GI complaints (34), and increased RPE (35). In line with this supposition, our findings showed that consuming 50 mmol/L sodium significantly reduced RPE, perceptions of thirst and GI discomfort compared to placebo. Trend analysis also showed that as the concentration of sodium increased, perceived thirst significantly decreased in a linear manner. Sodium consumption may increase osmotic material retained in the extracellaur space, increasing blood osmolality and thirst perception (36) The trials in this study provided sufficient fluid to increase pre- to post-session body mass with research suggesting thirst is stimulated following a 1–2% body mass loss (37, 38). Thus, taken together with previous research, the improved groundstroke performance induced by sodium consumption may have resulted from a combined attenuation of symptomologic distracters such as thirst, GI distress, and RPE instead of a direct physiological effect of hydration and warrants further investigation.

Despite the observed improvement in groundstroke performance, there was no evidence for an effect of sodium ingestion on serve performance. The reason for this is unclear but may be due to the fact that tennis serving is pre-planned and does not require the player to react to a stimulus, whereas the groundstroke task involved reacting to tennis balls being thrown in quick succession (2-s) by a LTA coach. The groundstroke task therefore encompassed greater cognitive components (including visual scanning, reaction time, and decision making), which may have improved through sodium ingestion.

Fluid lost during the control trial in this study (0.63 L/h, 0.9% of initial body mass) was mild compared to the range of mean fluid losses reported in the literature (0.6–2.6 L/h) (3). This is likely due to the indoor environment, the ambient temperature (16.5 ± 1.1°C), the duration of training sessions (60-min), and the protocol involving training and not match-play (39). It has been proposed that ≥2% fluid loss represents the threshold for reductions in endurance performance (32). However, impaired cognition has been observed at fluid losses of <1% (40, 41), which supports the assertion that the modest degree of hypohydration observed in the placebo condition (0.9% fluid loss) could have impaired cognitive function, probably as a byproduct of the aforementioned distracting symptoms, and subsequently reduced tennis skill.

Despite observing a statistically significant difference in UOsm between the 50 mmol/L sodium and control trials, there was no evidence for an effect of sodium ingestion on fluid loss (*p* = 0.08) or total sodium sweat loss (*p* = 0.37). Discrepancies between UOsm and fluid losses have been reported previously (42). Although evaluating fluid losses via changes in body mass aligns with previous research and is a suitable field-based technique (3, 5, 18, 20, 32, 43), there are sources of error associated with this method, including respiratory water loss and loss due to substrate exchange (22). Water produced during substrate oxidation, release of water previously bound with glycogen, and accumulation of water in the bladder also cause a dissociation between body mass changes and hydration status (22, 44). Thus, given the potential for error and the small fluid losses observed in this study, monitoring changes in body mass might not have been sensitive enough to detect subtle differences in hydration status between conditions.

There was no evidence of an effect of sodium ingestion on agility performance. Whilst limited data exist, previous research with soccer players suggests that hydration status does not influence 15-m sprint performance (30, 45). Thus, consuming sodium to mitigate the effects of hypohydration would not be expected to improve performance in the Pro-Agility test. It is important to note that the Pro-Agility test does not require players to react to a stimulus, which limits the cognitive requirements of the task. Hoffman et al. (46) reported improved lower-body reaction time when basketball players drank water during a 40-min game compared with no fluid (0 vs. 2.3% body mass loss). Hydration status might differently affect performance in pre-planned vs. reactive agility tasks. Alternatively, greater fluid loss than observed in this study (0.9% of body mass) and thus greater levels of hypohydration may be required to influence such tasks. Further research should aim to determine whether sodium ingestion improves reactive agility, which more closely replicates tennis match-play.

A limitation of this study was that research team members were not blind to the type of beverage that participants received, although the researchers strictly adhered to a pre-determined protocol. We used cluster randomization to randomize the order of the experimental sessions, with the unit of randomization being the whole sample of participants (order: 10, 50, 0, 20 mmol/L), which may have introduced systematic bias. However, there was no evidence of an order effect on the outcome measurements given that we found a linear dose response on UOsm and groundstroke performance as the dose of sodium increased from 0 to 10, 20, and 50 mmol/L. Although dietary sodium intake was measured, along with consumption of 250 ml of the sodium solution 20-min prior to testing, this may not have allowed enough time to affect pre-trial UOsm readings, affecting baseline hydration scores. Furthermore, whilst participants were blind to the sodium concentration of the beverages and the flavor was masked, we cannot guarantee that participants were not aware of the relative sodium content of the beverages, although participants anecdotally reported that they could not identify any differences between the drinks. Finally, this study also used a relatively small sample size (*n* = 12), which limits the precision of the effect estimates.

In conclusion, consuming 50 mmol/L of sodium before and during a 1-h tennis training session reduced UOsm and improved groundstroke performance in nationally-ranked tennis players. There was also evidence of dose-response effects, showing that ingesting greater sodium concentrations resulted in greater improvements in hydration and groundstroke performance. The observed enhancement in tennis skill may have resulted from an attenuation of symptomologic distracters associated with hypohydration, such as thirst, GI discomfort, and RPE. Practitioners and sports nutritionists should include sodium ingestion in their nutritional arsenal as a simple and effective strategy to improve tennis-specific skill.

## Data Availability Statement

The raw data supporting the conclusions of this article will be made available by the authors, without undue reservation.

## Ethics Statement

The studies involving human participants were reviewed and approved by Sports, Health and Exercise Science Ethics Committee at the University of Hull. The patients/participants provided their written informed consent to participate in this study.

## Author Contributions

EM, JB, ST, PM, and RV were responsible for the conceptualization and formulation of the overarching research question, as well as the development and design of study methodology. EM and JB were principally responsible for data collection. SO was responsible for data curation, statistical analyses, and writing the original draft of the manuscript. PM and RV were responsible for the overall oversight and leadership of the study. All authors reviewed, edited, and approved the final version of the manuscript.

## Conflict of Interest

The authors declare that the research was conducted in the absence of any commercial or financial relationships that could be construed as a potential conflict of interest. The handling Editor declared a past co-authorship with one of the authors RV.
